# Macroeconomic and environmental consequences of circular economy measures in a small open economy

**DOI:** 10.1007/s00168-021-01079-6

**Published:** 2021-09-25

**Authors:** J. Brusselaers, K. Breemersch, T. Geerken, M. Christis, B. Lahcen, Y. Dams

**Affiliations:** 1 Unit of Sustainable Materials, VITO, Boeretang 200, 2400 Mol, Belgium; 2 Department of Environmental Economics, Institute for Environmental Studies, VU University Amsterdam, De Boelelaan 1087, 1081 HV Amsterdam, The Netherlands; 3 Faculty of Economics and Business, KU Leuven, Vlamingenstraat 83, 3000 Leuven, Belgium

**Keywords:** C68, Q58, H20

## Abstract

This paper investigates the economy-wide impact of the uptake of circular economy (CE) measures for the small open economy (SOE) of Belgium, in particular the impact of fiscal policies in support of lifetime extension through repair activities of household appliances. The impact assessment is completed by means of a computable general equilibrium model as this allows quantification of both the direct and indirect economic and environmental impact of simulated shocks. The results show that different fiscal policy types can steer an economy into a more circular direction. However, depending on the policy type, the impact on the SOE’s macroeconomic structure and level of circularity differs. Furthermore, common claims attributed to a CE (e.g. local job creation or decreased import dependence) can be, but are not always, valid. Hence, policy-makers must prioritize their most important macroeconomic goals and opt for an according fiscal policy. Finally, this paper finds that the CO_2_ equivalent emissions calculated from a production (or territorial) perspective increase, while they decrease from a consumption perspective. This is explained by the substitution of international activities by local circular activities. This comparative analysis advocates for the consumption approach to assess the CE’s impact on CO_2_ equivalent emissions.

**Supplementary Information:**

The online version contains supplementary material available at 10.1007/s00168-021-01079-6.

## Introduction

The circular economy (CE) is gaining momentum as an alternative to traditional linear economic systems that rely on a take–make–dispose approach, e.g. as part of the European Green Deal (European Commission 2019). In this rationale, a CE introduces alternative flow models to an economic system that are cyclical and hence avoid waste to reduce negative environmental impacts (Ellen MacArthur Foundation et al. 2015) and avoid extensive use of materials. However, the widespread attention for CE has also resulted in a widespread of CE definitions. For example, a systematic analysis by Kirchherr et al. (2017) found 114 CE definitions, with some of the definitions confusing CE with recycling. However, recycling is just one option in a hierarchy of circular strategies (Allwood 2014). The framework by Potting et al. (2014), for example, includes no less than 10 different CE strategies, i.e. recover, recycling, repurpose, remanufacture, refurbish, repair, reuse, reduce, rethink, refuse. Recovery of materials and recycling are the least preferred options in this framework.

This paper presents a computable general equilibrium (CGE) model to investigate the economy-wide impact of the uptake of CE measures for the small open economy (SOE) of Belgium. Specifically, it assesses the impact of fiscal policies in support of repair activities to extend the lifetime of household appliances. In addition, the CGE also determines the environmental impact of the policies and increased circularity in terms of CO_2_ equivalent emissions.

As a hot topic, the CE has received ample attention in recent literature, but a meta-analysis by Kirchherr and van Santen (2019) on 160 papers still identified important flaws in the current body of literature. They discovered that 45% of these articles are conceptual, while ‘practitioners want empirical work that provides evidence on how to make CE work’. In addition, most empirical articles dependent on a limited number (< 10) of case studies which limits the external validity of these studies. Finally, large parts of the corpus (95%) focus on manufacturing industries, while only 9% focus on service industries (Kirchherr and van Santen 2019).

This is problematic for two reasons. First, services contributed 73.2% of the EU-28’s total gross value added in 2018 (EUROSTAT 2019a). Hence, in the context of the CE, the academic interest in the services sector is not proportional to the services’ overall economic importance. Second, many academics stress that services and service-based business models have the potential to play a crucial role in the envisaged uptake of the CE (Heyes et al. 2018; Korhonen et al. 2018; Kirchherr and van Santen 2019). Because of these flaws, the CE concept is currently loosely based on fragmented ideas from different scientific fields, including emerging fields and semi-scientific concepts (Korhonen et al. 2018), while most of the corpus (63%) emerges from natural sciences (Kirchherr and van Santen 2019).

The CGE model presented in this paper addresses several of the issues mentioned above. First, a CGE model bridges the gap between theoretical and empirical research (Franck 2013). The theoretical part of this paper’s CGE model mathematically describes circular activities and their interaction with linear activities. The empirical part consists of the calibration of the CGE model for the Belgian economy. Furthermore, the case of promoting repair activities based on empirical data evidently constitutes a service-based activity, addressing this gap in current literature. Second, the CGE model represents a holistic approach to assess the economy-wide impact of CE. This approach prevents fragmented conclusions and prevents the extrapolation of the conclusions from being hindered by small N. As such, this paper also complements earlier research by, for example, Brusselaers et al. (2019) who calculated the economic benefits of the repair of household appliances at individual consumer level. Third, the calibration of the CGE model introduces real data and captures the current economy’s structure. To our knowledge, this is the first time a service-based circular activity is fully integrated (and calibrated) in a CGE model and the first consistent split out of large economic sectors into detailed sectors that are relevant for CE activities at regional level (in particular maintenance and repair). Finally, the CGE is extended with a tool to assess the policies’ environmental impact in terms of CO_2_ equivalent emissions. This allows policy-makers to balance economic and environmental costs and benefits in the context of a CE, beyond recycling.

The use of CGE models is common practice whenever there is a need to quantify the effect of change in one part of the economy upon the rest as CGE models capture both the direct and indirect effect of simulated shocks (Freire-González et al. 2017). However, the use of CGE models in the context of the CE remains unexploited (Winning et al. 2017). Nevertheless, McCarthy et al. (2018) indicated that there is a need for a more macroeconomic analysis to provide understanding and clarity on the CE, for both public and private stakeholders. The few CGE models that do assess the CE focus on resource extraction or recycling (Sjöström and Östblom 2010; Winning et al. 2017). This is problematic for CE research in the sense that a truly circular approach would aim to reuse, repair or remanufacture whatever material and product possible, and only recycle what cannot be reused (Stahel 2016). In this rationale, recycling becomes one of the least preferred options in a hierarchy of material management strategies (Allwood 2014). This paper, for the first time, presents CGE modelling results with a focus on one of the more preferred circular strategies: lifetime extension through repair.

The remainder of this paper is structured as follows. Section 2 presents the methodology and related decisions while Sect. 3 presents the policy scenarios. Sections 4 and 5, respectively, present and discuss the results. Section 6 provides the conclusions of this research.

## Methodology

This paper is built around an existing and calibrated CGE model for the small open economy of Belgium. The following section briefly describes the basic CGE model’s characteristics. Subsequently, the following sections describe how circular activities are introduced in the model and how they are calibrated.

This paper opts for a CGE model because it is the most promising tool to simultaneously assess the economic and environmental impact of policies, compared to other available economic modelling tools such as input–output models, agent-based models or system dynamics models (Beaussier et al. 2019). While CGE models are widely accepted as useful tools for policy assessments and mathematically superior in representing market mechanisms, it is equally important to acknowledge the limitations of CGE models. First, the models depend on the chosen functional forms and parameters (Barker 2004). Second, experimental and behavioural economists criticize neoclassical assumptions such as rational behaviour and maximization of profit and utility (Yang and Heijungs 2017). Because of those assumptions, a CGE model might not be able to predict potential radical changes in preferences and technologies (Lahcen et al. 2020). However, this kind of radical changes are not part of this paper’s scope as the circular activities are calibrated based upon the actual economic structure.

### Basic CGE model

The initial model was used to assess recovery plans out of the COVID-19 crisis. Lahcen et al. (2020) and further developed and modified in this paper to allow an assessment of the uptake of the CE. The model is comparative static by nature and hence suitable for analysis of (fiscal) policy scenarios. This allows one to assess policy impacts, with little deviation in the economy’s underlying structure, compared to the baseline situation (following the rationale by Ciarli and Savona (2019)). The baseline situation in this rationale is the calibrated run of the CGE model, which reproduces the CGE model’s input data (e.g. the social accounting matrixes). Furthermore, the modified CGE model respects all standard general equilibrium assumptions part of the initial model and listed in Lahcen et al. (2020).

### Introduction of circular activities

Most CGE models tend towards highly aggregated sectors (Winning et al. 2017). The CGE model presented in this paper starts from a standard sectoral disaggregation into 12 basic sectors. The combination of these 12 sectors captures the entire economic structure. In addition, the CGE model distinguishes between 12 products/services, each relating to a specific production sector. These sectors and products/services are presented in Table 1 (subheading ‘Basic model’).

**Table 1:** Sectors and products/services present in the CGE model, after extraction of the circular sector and its conventional alternative

Sectors	Products/services
*Basic model*	
Agriculture, fishing, forestry	Agricultural products, fish, forestry products
Mining	Mining products
Industry	Industrial products
Energy	Energy
Construction	Construction products & services
Trade	Trade services
Land transport	Land transport services
Water transport	Water transport services
Air transport	Air transport services
Logistics and mail	Logistical services and mail
Market services sector	Market services
Non-market services	Non-market services
*Extended model*	
Circular: repair services sector (household appliances)	Circular: repair services for household appliances
	Related: spare parts household appliances
Conventional: retail new household appliances	Conventional: household appliances

None of the products or services are further defined. Household appliances encompasses all possible kinds of household appliances

The basic model’s sectoral disaggregation does not allow for the analysis of the envisaged circular strategy (i.e. lifetime extension through repair of household appliances). As stated by Winning et al. (2017), the analysis of specific circular strategies requires more detail. For this reason, two additional sectors are extracted and introduced in to the CGE model. First, the sector which repairs household appliances is extracted out of the 12 basic sectors. Second, the retail of new household appliances is extracted out of the 12 basic sectors and serves as the conventional alternative to the circular activity. The circular and conventional sectors provide substitutional products/services.

The extraction of these two additional sectors requires the extraction of additional (competing) products and services. First, the ‘repair of household appliances’ services is extracted and subsequently added as a separate service to the CGE model, produced by the circular activity (Table 1, subheading ‘Extended model’). However, consumers are not obliged to repair their household appliances and can also purchase new. For this reason, ‘Household appliances’ are extracted and added as a product to the CGE model. Finally, ‘Spare parts of household appliances’ are extracted and separately introduced in the CGE model as it is assumed that the circular repair services will make extensive use of this intermediate product. All other intermediate products and services applied by the repair activities remain captured by the basic product/service classification.

As the circular repair services and more conventional retail of new household appliances satisfy the same consumer need, their relationship can be modelled according to Keller (1975) who maximizes utility $x_{0}$ by means of the nested CES utility function:

1 $${\text{Max }}x_{0} = \left[ {\alpha_{1}^{{\frac{1}{\sigma }}} \left( {x_{1} - \overline{x}_{1} } \right)^{{\frac{\sigma - 1}{\sigma }}} + \alpha_{2}^{{\frac{1}{\sigma }}} \left( {x_{2} - \overline{x}_{2} } \right)^{{\frac{\sigma - 1}{\sigma }}} } \right]^{{\frac{\sigma }{\sigma - 1}}}$$

Hence, welfare is determined by comparison of the current purchased volumes of each utility component $x_{i}$ and the subsistence levels of the utility components $\overline{x}_{i}$. In this simplified case, we represent the utility calculation for two contributing utility components (i.e. $i = 1, 2$). The weight of each component is determined by the distribution parameters $\alpha_{i}$ (with $\sum\nolimits_{i} {\alpha_{i} = 1}$) and the elasticity of substitution $\sigma$ (with $0 \le \sigma < \infty$). Due to a lack of information on the elasticity between the conventional and the circular alternatives, this elasticity is initially set at 3.35. This value equals the average elasticity of substitution between domestic and imported varieties for a basket of goods from the GEM-E3 model (Capros et al. 2013). Albeit conservative, this is a solid assumption for the elasticity between two substitute goods/services which aim to satisfy the same consumer need. The robustness of the assumption was checked by means of a sensitivity analysis at second stage in which the elasticity is decreased to lower levels. Hence, this sensitivity analysis assesses the impact of the assumption of perfect versus imperfect substitution. Finally, maximization of $x_{0}$ is subject to budget constraint:

2 $$p_{1} x_{1} + p_{2} x_{2} \le y_{0}$$

with the utility components’ prices $p_{i}$ and available budget $y_{0}$. Determining the optimality conditions for this problem entails the following result:

3 $$x_{0} = \frac{{y_{0}^{d} }}{{p_{0}^{1 - \sigma } }}\left[ {\alpha_{1} p_{1}^{1 - \sigma } + \alpha_{2} p_{2}^{1 - \sigma } } \right]^{{\frac{\sigma }{\sigma - 1}}} = \frac{{y_{0}^{d} }}{{p_{0}^{1 - \sigma } }} p_{0}^{ - \sigma } = \frac{{y_{0}^{d} }}{{p_{0} }}$$

Here, $y_{0}^{d} = y_{0} - p_{1} \overline{x}_{1} - p_{2} \overline{x}_{2}$, and $p_{0} = \left[ {\alpha_{1} p_{1}^{1 - \sigma } + \alpha_{2} p_{2}^{1 - \sigma } } \right]^{{\frac{1}{1 - \sigma }}}$. This mathematical problem, also leading to the price setting for the substitute goods, is completed for two substitutable goods in a separate node of the nested function (see Fig. 2 in Appendix), devoted to consumer choices on household appliances (i.e. repair or acquire a new household appliance). Applying this approach allows to consistently introduce the circular activities into the CGE model. The calibration of the model is based upon the current activity level in the circular sector and represents the actual circular sector’s structure (see following section). Since this approach acknowledges that circular activities are already taking place, the Stone–Geary preferences (i.e. introducing a minimum level of demand for repair activities as a fraction of the Social Accounting Matrix’s (SAM) demand for repair activities) are also applied for the new circular sector, just like for all other sectors.

### Data extraction and calibration

The approach described in the previous section requires a consistent split out of large sectors into more detailed sectors in a SAM for the Belgian economy. This SAM is subsequently used as input data for calibration. The construction of this SAM combined several different data sources. At first, the 2015 version of the Belgian Supply and Use Tables (SUTs) provide monetary information on the supply and use of goods per sector. The original SUTs are published by the Belgian Federal Planning Bureau (2018) and contain 64 industries and 64 product groups. As mentioned above, the SUT data are aggregated to a limited number of sector and products groups. Subsequently, the data are merged with the annual account aggregate data. However, the use of different methodologies in the construction of the SUT, on the one hand, and the national accounts, on the other, leads to some discrepancies that need to be addressed. The largest discrepancy results from the use of a domestic concept of trade in the construction of the SUT, while the national accounts apply a national concept. To align the SUT with the national accounts, the existing discrepancy is divided across the different goods according to the share of the relevant products in total imports or exports in the SUT. A similar approach is used for discrepancies between taxes and subsidies in the national accounts and the SUT, following the approach of EUROSTAT (2008) and of the OECD (2006). The result is a SAM matrix where the linkages between supply and use of products/services and the institutional sector accounts are reported in a consistent methodology.

The aim of this paper is to assess the impact of the uptake of CE measures. Therefore, prior to the aggregation of the SUT sector and product groups, two industries and three product groups closely related to the CE were split up using additional data sources. The purpose here was to isolate certain industries and products of particular relevance to the CE. Specifically, we isolate the retail trade of new household appliances from the general retail trade industry (NACE 2 rev.47), and the repair of household appliances is isolated from the repair of computers and consumer goods (NACE 2 rev. 95). As such, the SAM distinguishes the current conventional (and predominantly linear) retail activity from the circular repair activity.

To isolate the retail trade of new household appliances from the general retail trade industry, we rely on data from Eurostat’s Structural Business Statistics (SBS). Specifically, we collect data on the sales of the CPA 470,054 ‘Retail trade services of electrical appliances’, which we use as a proxy for output. Next, we assume the same supply and use structure as that of the general retail trade industry, which is rescaled according to the relative size of CPA 470,054 to the retail trade industry (NACE 47). We proceed similarly using available SBS data to isolate the repair of household goods from NACE 2 rev.95. Finally, the isolation of the retail trade and repair industry as well allowed for calibration of the CGE model.

On the product side, we divide manufactured electrical goods into household goods excluding spare parts, on the one hand, and manufactured spare parts of household appliances, on the other hand. To estimate the total amount of both products produced, we use the import and export flows of household appliances and the parts thereof as well as the total consumption of these goods in Belgium obtained through the Household Budget Survey (Statistics Belgium 2018). With these numbers at hand, it is possible to obtain an estimate of the domestic production of each type of good. Trade margins, taxes and subsidies are assumed to be similar in relative size to those of the general electrical goods. The supply structure is equally assumed to be of a similar nature to that of the general electrical goods. For the use table, it is assumed that the household appliances are mostly used for final consumption. Only a small fraction is used by the manufacturing sector according to the share of general electrical goods used by the manufacturing industry. Similarly, the use of spare parts for household appliances is assumed to be mostly destined for final consumption. Again, a fraction is used by the manufacturing industry of general electrical goods. The remaining fraction used by domestic industry is assigned to the repair of household appliances industry, which we isolated in an earlier step. The same approach is applied to isolate the repair (of household appliances) services from the market services. The estimate for the use of these services is based upon the proportional importance of labour demand by the repair of household appliances sector in relation to labour demand by the market services sector.

Disaggregation of these sectors, products and services allows for the assessment of the impact and structure of the CE measures in comparison to the more linear business-as-usual scenario in the reference year. In addition, the disaggregation allows the repair (circular) and conventional (more linear) activities to be modelled as substitutable consumption products. This rationale allows for the design of policy scenarios in support of the CE, and the assessment and comparison of these scenarios’ results.

## Policy scenarios

CGE models computationally derive the impact of policy at economy-wide level. Following the calibration of the CGE model (based on the developed SAM with inclusion of the extracted circular sectors, products and services), the model is run a first time to compute the baseline scenario, which represents the economy’s pre-policy situation. As the model is calibrated by making use of 2015 data, the baseline represents the structure of the Belgian economy in 2015.

This paper investigates the impact of fiscal policies and taxation as Stahel (2016) described how this type of policy can recoup costs ‘through remarketing rather than scrapping materials’. This rationale corresponds to the lifetime extension through repair. However, the fiscal policies can either aim to promote sustainable practices or discourage unsustainable practices. This paper proposes four different policy scenarios to cover all possible fiscal options:

- *Restrictive fiscal policy* increased taxes to discourage the consumption and production of unsustainable products and services. The tax rate on linear purchases of new household appliances via the retail sector is increased in a stepwise process. Specifically, the original tax rate on new (linear) purchases is repeatedly increased by 0.3 percentage points. The increase is repeated 15 times to assess the economy’s evolution.
- *Expansionary fiscal policy* taxes are cut to encourage the consumption of sustainable (i.e. circular) products and services. The original tax rate on circular repair services is repeatedly (15 times) decreased by 0.3 percentage points. The evolution of the economy is monitored throughout this stepwise process.
- *Hybrid fiscal policy* combination of the restrictive fiscal policy and the expansionary fiscal policy. This results in a simultaneous decrease in the tax rate on repair services (− 0.3 percentage points) and tax rate increase on new purchases (+ 0.3 percentage points). Both policy types can address different sectors simultaneously to assess their combined impact.
- *Generic green fiscal reform* a tax cut (− 0.3 percentage points) on all services in addition to the restrictive fiscal policy on retail of new household appliances (+ 0.3 percentage points). This kind of tax cut on services is advocated by numerous stakeholders (e.g. Meuleman and Parker (2014) and European Environmental Bureau (2017)) as a promising tool to stimulate the uptake of the CE as the tax cut favours trade in services instead of trade of materials and products.

## Results

The presented CGE model is comparative static by nature and hence does not introduce dynamics or future developments. This allows the impact assessment of different policy options, with little deviation in the economy’s underlying structure, compared to the baseline scenario (following Ciarli and Savona 2019). As the outcome of a static CGE model, the baseline scenario basically reproduces the original SAM (used as input data for the CGE’s calibration). The following sections present the results of the comparative analysis.

### Economic results

**Fig. 1:**
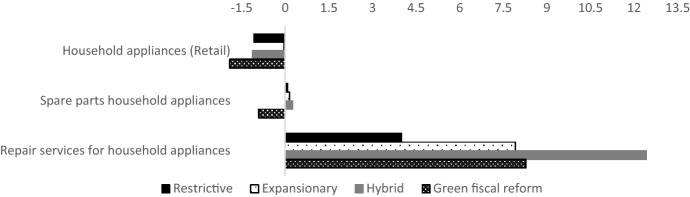
Percentage change in traded volumes per product and sector following the maximum tax increase or decrease allowed per policy scenario, targeted sectors. *Note* Targeted sectors are ‘Household appliances (retail)’, ‘Spare parts household appliances’ and ‘Repair services for household appliances’

**Fig. 2:**
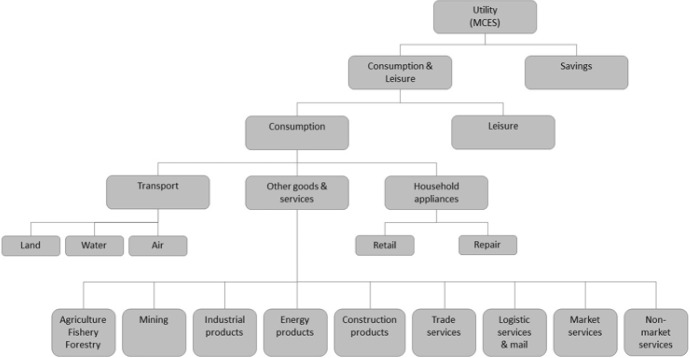
Generic structure of the consumers’ utility functions

The CGE model reports on the impact of each fiscal policy on the traded volume of each individual product and service (i.e. all purchases of these goods and services). Figure 1 presents the direct impact of the policies on the targeted products and services, i.e. the retail of household appliances, spare parts for household appliances and repair services for household appliances. The impact is expressed as a percentage change in traded volume by comparing the baseline situation to the final simulation. In the final simulation, the CGE model assumes that the tax increase or decrease reaches its maximum or minimum level, respectively, at the end of the stepwise tax adaptation (i.e. plus or minus 4.5 percentage points).

As shown in Fig. 1, all fiscal policies manage to stimulate the repair sector for household appliances. However, the restrictive tax policy is least effective in stimulating repair activities (+ 4% in traded volume compared to the baseline situation), while the hybrid fiscal policy (the combination of additional tax on linear activity and decreased tax on circular activity) is most effective (+ 12% in traded volume compared to the baseline situation).

Simultaneously, all tax policies manage to discourage the conventional retail activities (new sales), albeit in different magnitudes. Retail of new household appliances is least affected by the expansionary tax policy (decreased traded volume of 0.03%) and most affected by the green fiscal reform policy, which manages to decrease sales by 1.9%. The impact on the spare parts follows the evolution of the impact on the repair services in the restrictive, expansionary and hybrid scenario. This is not the case in the green fiscal reform scenario, as this policy does not solely target the repair sector, but favours other sectors as well.

In addition to the directly targeted products and services, the policies also indirectly impact all other products and services. These indirect impacts are presented in Table 2, per fiscal policy. The indirect impact is again expressed as the percentage change in traded volumes by comparing the baseline situation with the final simulation.

**Table 2:** Percentage change in traded volumes per product and sector following the maximum tax increase or decrease allowed per policy scenario

Policy scenario Product/service	Restrictive	Expansionary	Hybrid	Green fiscal reform
Agricultural products, fish, forestry products	+ 0.0275	− 0.0005	+ 0.0270	− 2.2808
Mining products	+ 0.0214	− 0.0004	+ 0.0210	− 1.9661
Industrial products	− 0.0004	− 0.0001	− 0.0006	− 0.3153
Energy	+ 0.0153	− 0.0003	+ 0.0151	− 1.1868
Construction products & services	− 0.1803	+ 0.0017	− 0.1786	+ 10.7908
Trade services	+ 0.0071	− 0.0002	+ 0.0069	+ 1.0319
Land transport services	+ 0.0263	− 0.0004	+ 0.0259	− 1.9108
Water transport services	+ 0.0324	− 0.0005	+ 0.0319	− 1.9556
Air transport services	+ 0.0127	− 0.0003	+ 0.0124	+ 0.1328
Logistical services and mail	+ 0.0339	− 0.0005	+ 0.0333	− 1.6596
Market services	− 0.0044	− 0.0001	− 0.0045	+ 2.4855
Non-market services	+ 0.0047	− 0.0001	+ 0.0046	− 0.6719
Household appliances (retail)	− 1.0763	− 0.0331	− 1.1104	− 1.9020
Spare parts household appliances	+ 0.0860	+ 0.1552	+ 0.2510	− 0.9085
Repair services for household appliances	+ 4.0092	+ 7.9122	+ 12.4206	+ 8.2737

All numbers represent percentage changes. These changes are given to four decimal places to correspond with the importance of the targeted sector (i.e. repair of household appliances) in the current economy (i.e. it accounts for 0.004 per cent of the current GDP)

In the hybrid and restrictive scenarios, the traded volumes increased for all products and services, except for the industrial products, construction products and services, and the market services. The opposite situation is observed for the expansionary scenario: the traded volume of the non-targeted products and services decreased, except for the construction sector. Finally, the green fiscal reform scenario’s impact on the non-targeted products and services is most comparable to the expansionary scenario’s impact. Most traded volumes decreases, except for the market services, air transport services, trade services and construction products and services. For the latter category, the magnitude of change (+ 10.79%) surpasses the impact in the expansionary scenario (+ 0.0017%) considerably, however. The considerable impact in the green fiscal reform scenario is explained by the observation that the construction sector makes relatively more use of services compared to other sectors. As the green fiscal reform favours all types of services, this allows the construction sector to benefit most.

While Table 2 represents the impact of the fiscal policies on the traded volumes for each product and service, Table 3 presents the policy impact on a selection of the generic (macro)economic parameters present in the CGE model. At first, the household income is positively affected by the expansionary and green fiscal reform policies (respectively, + 0.0003% and 2.4123%), but negatively affected by the restrictive and hybrid policies (respectively, − 0.0274% and − 0.0271%). This is a straightforward consequence of the policies’ design which is built around encouraging or discouraging consumption.

**Table 3:** Change in macroeconomic parameters following the maximum tax increase or decrease allowed per policy scenario

Policy Scenario	Economic parameter (percentage change)									Part of economy impacted (% of GDP)
	Household income	Investment	Government Budget	Household Labour supply	Logistics	Origin of products and services			GDP	
						Belgium	EU	ROW		
Restrictive	− 0.0274	− 0.2772	− 0.0356	+ 0.0062	+ 0.0295	+ 0.0040	− 0.0430	− 0.0426	− 0.0270	0.0510
Expansionary	+ 0.0003	+ 0.0028	+ 0.0005	− 0.0005	− 0.0005	− 0.0008	+ 0.0003	+ 0.0002	+ 0.0004	0.0012
Hybrid	− 0.0271	− 0.2774	− 0.0351	+ 0.0064	+ 0.0289	+ 0.0039	− 0.0427	− 0.0425	− 0.0266	0.0513
Green fiscal reform	+ 2.4123	+ 14.5548	+ 2.4123	+ 0.1040	− 1.4340	+ 0.3936	+ 2.3487	+ 2.2574	+ 1.5672	4.5005

All numbers, except in the last column, represent percentage changes. These changes are given to four decimal places to correspond with the importance of the targeted sector (i.e. repair of household appliances) in the current economy (i.e. it accounts for 0.004 per cent of the current GDP). The parameter ‘Part of economy affected’ is calculated by taking the sum of the absolute values of the changes in sectoral activity in relation to the GDP

The evolution of household income is not related to the job creation. The households provide more labour in the restrictive, hybrid and green policy scenario (respectively, + 0.0062%, + 0.0289% and + 0.1040%) while they provide less labour in the expansionary scenario (− 0.0005%). Hence, the restrictive and hybrid policies manage to create more jobs but offer lower wages. Note that the economy’s production processes’ demand for logistical services evolves in line with the evolution in labour requirements per policy scenario (albeit with a higher magnitude of change is higher in the logistical services requirements).

In accordance with the policies’ impact on household income level, the restrictive and hybrid policy scenarios are characterized by a decrease in the investment level (respectively, − 0.02772% and − 0.2774%), a decreased government budget (respectively, − 0.0356% and − 0.0351%) and a decreasing gross domestic product (GDP) (respectively, − 0.0270% and − 0.0266%). The magnitude of change for these three parameters does not differ considerably among these two policy scenarios. On the other hand, the expansionary and green fiscal reform policies manage to achieve a growth in the investment level (respectively, + 0.0028% and + 14.5548%), government budget (respectively, + 0.0005% and + 2.4123%) and GDP (+ 0.0004% and + 1.5672%). However, the magnitude of change differs considerably for these policies; the green fiscal reform has a much more elevated impact on these three parameters.

Note that the policies’ overall impact on the available government budget consists of both a direct and an indirect part. The direct impact relates to the policies properties (i.e. taxes generate revenues while subsidies generate expenditures). However, this direct impact is partially levelled out by an indirect impact. This is explained by the CGE model’s assumption that the government’s utility level is fixed compared to the baseline situation. Hence, the direct impact of tax incomes is compensated by shifts in other governmental revenues and expenditures. Because of this reason, the policies’ overall impact on the government budget strongly correlates to the policies’ impact on GDP (as the government’s revenues stem from a taxation on economic activities).

In addition, it is worthwhile considering the origin of the products and services that are traded in this SOE. The restrictive and hybrid policy increase the importance of domestically produced products and services (respectively, + 0.0040% and 0.0039%), at the expense of products imported from the EU (respectively, − 0.0430% and − 0.0427%) or the ROW (respectively, -0.0426% an − 0.0425%). Again, the magnitude of change in the origin of the traded products and services is highly comparable for the restrictive and hybrid policies. The expansionary policy, on the other hand, increases the importance of products and services imported from the EU (+ 0.0003%) and the ROW (+ 0.0002%), while domestically produced products and services are traded less (− 0.0008%) within the SOE. These finding on import dependence are in line with common economic theory. The green fiscal reform policy finally increases the traded volume of products from all origins.

The penultimate column in Table 3 shows an indication of the extent to which the SOE was affected by the fiscal policies (and their accompanying uptake of the CE). This indication is a result of the sum of the absolute values of the changes in each of the traded products and services’ contribution to the GDP. It can be observed that the expansionary policy impacts the economy the least. Aggregation of the changes in each traded product and each traded service’s contribution to the GDP only amounts to 0.001% of the SOE’s GDP. The green fiscal reform policy on the other hand manages to impact the economy the most (by around 4.5%). The restrictive and hybrid policies again have a comparable impact and manage to generate an impact of 0.05% of the economy. The final column in Table 3 is discussed in the following section.

### Environmental impact

Since the different policies affect the economy differently, it can also be expected that the policies entail a different environmental impact. The environmental impact is estimated by means of the CO_2_ equivalent emissions. However, these emissions are calculated using two different perspectives: the production perspective and the consumption perspective.

Following the EEA (2013), the production perspective accounts for emissions from economic activities of a country’s resident companies and private households in relation to their output (i.e. including emissions related to the country’s exports). Likewise, the EEA (2013) describes how the consumption perspective accounts for emissions from a country’s final consumption, irrespective of the geographic location where the production of the goods and services takes place. Hence, emissions for production of exported goods and services are not considered while emissions for imported goods and services are added to the calculation.

Table 4 presents the environmental impact of the different policies compared to the baseline scenario. Some observations are apparent. First, the production and consumption perspective appear to find opposing trends per policy scenario. In case the production perspective finds increasing (decreasing) emissions for a policy scenario, the consumption perspective will find decreasing (increasing) emissions for that same scenario. Second, the emissions calculated from a production perspective tend to increase for all scenarios while they decrease when calculated from the consumption perspective. Put differently, the territorial emissions, generated within the country increase in the restrictive, expansionary and hybrid scenario but the global emissions related to domestic consumption decrease. The only exception to this rationale is the Green Fiscal Reform scenario. Third, the restrictive and hybrid policy scenarios have comparable impacts in both perspectives. The expansionary scenario, however, entails the smallest impact when calculated from a production perspective while it entails the largest impact when calculated from a consumption perspective compared to all other scenarios (leaving aside the Green Fiscal Reform scenario).

**Table 4:** Percentage change in CO_2_ equivalent emissions following the maximum tax increase or decrease allowed per policy scenario, compared to the baseline scenario

Policy scenario Perspective	Restrictive	Expansionary	Hybrid	Green Fiscal Reform
Production	0.0334	0.0205	0.0334	− 2.0900
Consumption	− 0.0492	− 0.0636	− 0.0492	0.6826

These findings tend to suggest that it is preferable to assess emissions by means of the consumption approach, as this approach provides a complete picture on a region’s contribution to global emissions. The production approach on the other hand only provides a partial view on these emissions. However, limited data availability often prevent the use of the consumption perspective, as data on emissions abroad are not always available. (Athanassiadis et al. 2018).

### Sensitivity analysis

The assumed elasticity of substitution was subject of a sensitivity analysis to assess the impact of this parameter on the CGE’s results. This robustness check introduces a stepwise decrease in the initial elasticity of substitution (3.35) to a minimum level of 0.9. This minimum level is determined as 0.9 is also the elasticity of substitution between household appliances and other goods in the model (value taken from the GEM-E3 model described by Capros et al. (2013). The robustness check demonstrates an insignificant impact on the macroeconomic indicators (e.g. GDP). This is result of the model’s extensiveness which leads to balancing trends due to minor changes.

The impact of the elasticity on the policy’s effectiveness in terms of encouragement (discouragement) of the circular (conventional) product is more distinctive and linear. A stepwise decrease in the elasticity of substitution to a minimum level of 0.9 resulted in a lower but limited decrease in the consumption of new household appliances in both the restrictive and expansionary scenario. In case the minimum elasticity of substitution is reached, the consumption of new household appliances is 0.024% and 0.033% above the consumption levels in case of the initial elasticity of substitution for the restrictive and the expansionary scenario, respectively. It is especially the consumption of repair services which is impacted. In case of the minimum elasticity of substitution, the consumption of repair services is 3.8% and 5.16% below consumption levels in case of the initial elasticity of substitution for the restrictive and the expansionary scenario, respectively. Hence, in particular the consumption levels of the circular alternative appear sensitive to the chosen elasticity of substitution. However, due to the limited importance of this activity (e.g. as part of the GDP), the overall economic trends and impact of the proposed policies are not affected by changing levels of substitution between the conventional and circular activities.

## Discussion

### Magnitude of the economic impact

The changes in traded volume per product or service (Table 2) and the changes in the macroeconomic parameters (Table 3) might appear to be of negligible importance. However, putting these numbers in perspective demonstrates that the fiscal policies and the increased circularity have a disproportionally large impact on the economy. This is illustrated by two factors. First, the isolated products and services account for limited contributions to the GDP: only 0.004% of Belgian GDP can be attributed to the repair of household appliances; hence, this remains a niche activity at present. Accordingly, the trade in spare parts for household appliances also accounts for a rather limited share of the GDP (0.023%) and the traded volume in (new) household appliances contributes 0.483% to GDP. Second, the imposed shocks are the result of fiscal policies that, at most, increase or decrease the existing tax rates by 4.5 percentage points and hence entail even smaller changes in consumption prices.

The economic impact of the fiscal policy and the uptake of the CE is remarkable because of the limited magnitude of the imposed shock and the limited economic importance of the targeted repair sector. While the policies manage to increase the repair activities of household appliances by 4.00% to 12.42%, their impact indirectly trickles down to all other sectors. As a result, the aggregate impact surpasses the size of the repair sector for household appliances by twelve times and changes the SOE’s GDP considerably. Thus, the generic impact of the policies outweighs the relative economic importance of the niche repair sector for household appliances.

At product/service level, the restrictive and hybrid policies manage to increase or decrease the traded volume for all products and services (except industrial products) by more than 0.004% (which is the proportional economic importance of the repair services in the SOE). The expansionary policy has the smallest impact on the traded volumes of all products and services while the green fiscal reform policy has the largest impact. This is a consequence of the latter policy’s broader scope, which goes beyond the retail, repair and production of service parts for household appliances sectors.

Apart from the repair and retail of new household appliances and the spare parts of household appliances, the products and services whose traded volume is affected the most by the policies, in decreasing order of magnitude, are: ‘Construction products and services’, ‘Logistical services and mail’, and ‘Water transport services’. This indicates that the construction sector is more responsive to changes in the retail sector, while the water transport, logistics and mail sectors are more responsive to the repair sector.

### Increased circularity

The expansionary policy is more positive in nature as it aims to encourage circular activities. For this reason, this policy also manages to achieve a higher level of circularity (measured in terms of traded repair services) in comparison to the purely restrictive policy. On the other hand, the expansionary policy is least effective in discouraging sales of new household appliances (i.e. the more linear alternative). To decrease the undesirable linear activities, it appears necessary to also implement a discouraging and restrictive tax regime. Note, however, that the restrictive policy tends to decrease the GDP (albeit simultaneously increasing the households’ labour supply). Therefore, in the current set-up, a trade-off is required between the ambition to limit unwanted environmental externalities and the need to maintain (linear) economic activity. Alternatively, governments can also aim to increase consumers’ interest in repair services of household appliances as opposed to the purchase of new appliances. In the CGE model, this would manifest itself in increased cross-price elasticities between repair and retail and an upsurge in the extent to which consumers switch to repair following price increases in the retail price of new appliances.

The hybrid policy, like the restrictive policy, is characterized by decreasing economic parameters (e.g. government budget, GDP, investments and household income). Hence, the restrictive element in the hybrid policy is not fully compensated by the expansionary elements.

The green fiscal reform manages to increase the repair activities more than the purely expansionary policy targeted at the repair sector. So, it appears as if there is an element of truth in the claim to cut taxes on services in order to stimulate the CE as suggested by Stahel (2016) or the European Environmental Bureau (2017). Yet, the hybrid policy managed to increase the circular activities more. This is explained by the observation that part of the green fiscal reform benefits is used for different purposes than the uptake of the CE (e.g. construction activities increase by 10.76%). Those other purposes make economic sense, as GDP manages to grow most in the green fiscal reform scenario, but they do not necessarily align with the circular objectives. Hence, it appears recommendable to apply a hybrid policy and focus fiscal efforts on the desired circular activities and unwanted linear activities.

However, the extent to which the policies increase (decrease) the circular (linear) activities differs considerably. On the one hand, expansionary fiscal policies are more effective in the promotion of circular activities compared to restrictive fiscal policies. On the other hand, the restrictive fiscal policies are more effective in reducing the existing linear activities. Policy-makers should reflect on their priorities: encourage circularity or discourage less circular activities.

In addition, the analysis demonstrates that the impact of the different fiscal policies is considerable but not sufficient to achieve a full circularity. Achieving full circularity will require a more holistic approach and a set of accompanying policies in addition to fiscal policies. As such, the paper provides empirical evidence for findings by Hartley et al. (2020) and Nohra et al. (2020). They advocate for circular procurement, robust standards and norms in production, liberalization of waste trade, support for industrial parks and awareness campaigns in addition to fiscal policies in support of the CE. All of these policies aim to promote circular products and services over conventional products and services. However, there is also a need to develop circular products and services. For this reason, Blomsma et al. (2019) recommend policies in support of the early stages of CE oriented innovations.

### Job creation and import dependence

The CE is increasingly perceived as stimulus for local job creation (Stahel 2016; Geissdoerfer et al. 2018). This paper provides a quantified sectoral and economy-wide indication of the effects on jobs, depending on the type of fiscal policy chosen. The expansionary policy does increase circular activities, but simultaneously decreases the overall labour supply by households. All other policy types, however, do stimulate job creation, albeit to different extents. Disaggregation of the overall labour requirement into sectoral labour requirements provides additional insights in this evolution (see Table 5 in Appendix). In all policy scenarios, the increase of jobs in the circular sectors (repair of household appliances and the related spare parts of household appliances sector) occurs at the expense of a decreased labour requirement in some other sectors. Obviously, the number of jobs in retail of new household appliances is affected the most. In the restrictive and hybrid policy scenarios, most sectors generally experience an increased labour demand, except for the construction sector. The increased labour demand by all other sector is a result of the restrictive policy’ nature: it limits activity in the conventional, more linear sector. Consequently, economic resources are redirected towards other sectors, allowing them to grow. The expansionary policy tends to have an opposite impact on sectoral labour demand. As this policy encourages circular activities, it deprives resources from the conventional sectors and redirects them into the circular sector. Ultimately, this paper confirms an often made claim: a CE, supported by fiscal policy, can create jobs under specific conditions and at the expense of job losses in existing sectors.

In any case, the analysis shows that a transition towards a more CE will simultaneously lead to job creation in some sectors and job destruction in other sectors. Governments can anticipate this process and avoid unemployment because of a discrepancy between the available and the required skills on the labour market. This can be achieved by proactively providing education and training and encouraging inter-sectoral labour movement. The latter policy recommendation supports the findings by Burger et al. (2019) who claim that the future development of a CE will require specific training and education programmes. In addition, a lack of expertise and skills is considered as an important barrier to the uptake of the CE (Rizos et al. 2016). The education and training programmes can address that barrier.

Advocates of the CE also claim that it decreases a nation’s import dependence as more resources remain present in its economy (see, for example, Wijkman and Skånberg (2015), Van Buren et al. (2016) and Geissdoerfer et al. (2018)). This claim is confirmed in the restrictive and hybrid fiscal policy scenarios. Following these policies, the traded volume of products and services imported from the EU and the ROW decreases. Simultaneously, more domestically produced products are traded. It is difficult, however, to attribute this entirely or solely to circularity given that, depending on how fiscal policies are implemented, they can affect national accounts differently, regardless of their desired aims. This becomes apparent in the expansionary fiscal policy scenario, which is characterized by and increased traded volume of imported products and services. Thus, a CE can decrease a region’s import dependence, but not in all circumstances and in each policy scenario. In case a policy in favour of increased circularity strives for decreased import dependence, it needs to simultaneously (and considerably) decrease the linear economic activities.

### Environmental impact

The different policy scenarios entail a different environmental impact. This is a straightforward conclusion following the observation that the different policies also entail a different economic impact. However, most apparent are the differences between the calculated emissions from a production perspective and a consumption perspective. On the one hand, the production perspective indicates that emissions within a country’s territory increase because of the uptake of circularity. This is consequence of the increased local economic activities in these policy scenarios. On the other hand, the consumption approach demonstrates that emissions related to the country’s consumption decrease at global level. Hence, the increased economic activities within the country’s territory manage to emit less compared to the economic activities which disappeared at global level. This provides important insights for policy-makers seeking for a balance between environmental and economic policy gains. From a production point of view, one can conclude that economic gains occur at the expense of increased emissions. However, accounting for emissions abroad (both imported and exported), the analysis demonstrates that the economic gains related to increased circularity also achieve environmental gains at global level.

This observation calls for a careful analysis of the environmental impact of policies which aim to increase an economy’s circularity levels, especially because it can be expected that increased circularity encourages local economic activities within the economy. The more holistic consumption perspective is likely to provide a more robust assessment of an economy’s contribution to climate change. This finding fits in a dynamic discussion on this topic and conflicts with earlier research findings on the need for a consumption perspective by, for example, Barrett et al. (2013). This conflicting conclusion can be explained by pointing at the open character of the considered SOE of Belgium. This openness increases its dependence on emissions in regions abroad, as confirmed by Athanassiadis et al. (2018).

### Limitations

The presented analysis is prone to some limitations. First, the model departs from, and maintains, the current SOE’s structure and mechanisms. The fiscal policies are not supported by any other transitionary policy in support of the CE. Consequently, the competitiveness of the CE sector over its conventional (linear) counterpart in this CGE model is not strengthened by more structural measures (e.g. prohibitive legislation on emissions or material use or support for research and development). This rather conservative approach cautions against hasty conclusions and results in a moderate increase in the circularity. In reality, however, the uptake of the CE most likely requires a more integrated and comprehensive policy framework (Lieder and Rashid 2016; Homrich et al. 2018).

Second, the analysis presented in this paper has been validated by running a robustness check on the assumptions (e.g. the elasticities of substitution). This robustness check confirmed all findings, but no further sensitivity is run on external parameters. These external parameters might impact the responsiveness of the economy to the CE and supportive fiscal policies. Important examples of external parameters are the price stickiness and the monetary policy conduct (Annicchiarico and Di Dio 2015). Note as well that the elasticity of substitution between circular and less circular activities is likely to become more important when that substitution effect is investigated in more detail (and isolated from other trends in the economy). Future research could further investigate the relationship between circular consumption and less circular consumption. Also, the analysis of consumer attitude and purchase intention towards circular products and services can contribute to this topic.

Third, the CGE model built adheres a purely economic approach. It does integrate the environmental aspect of CO_2_ equivalent emissions, but no other environmental impacts are considered (e.g. land and water use). For an overview of CGE models and other types of models with the inclusion of environmental aspects (albeit from the point of view of structural economic change), see Ciarli and Savona (2019).

Fourth, it is likely that new household appliances are more energy efficient compared to old appliances (Trotta 2018; Bhadbhade et al. 2020). In this rationale, energy efficient appliances indirectly reduce CO_2_ emissions related to energy consumption. This paper neglects that possibility and assumes constant energy efficiency. That simplification is justified by the fact that the SAM used for calibration is built around real consumption levels of both energy and new household appliances. Hence, these data already account for improved energy efficiency of the current generation of household appliances. The analysis did not attempt to assess the impact of improved energy efficiency for the future generation of household appliances due to a lack of data on future improvements. In addition, increased energy efficiency is likely to be partially offset by rebound effects because households, for example, acquire more appliances per household or use their appliances more intensively (Fullerton and Ta 2020; Bhadbhade et al. 2020). Well-considered energy efficiency standards can help to counterbalance those rebound effects (Fullerton and Ta 2020).

Finally, this paper presents a static comparative CGE model. Future research could investigate the possibilities of dynamic models to investigate the impact of circular options. Note that the relevance of the presented static and comparative analysis is not affected by the proposal to introduce the aspect of time in future models. This paper’s analysis nevertheless manages to provide insights in to the economic mechanisms behind a transition towards more circularity. This provides essential information to, for example, policy-makers.

## Conclusion

Much is said about the CE. It is supposed to decrease a nation or region’s import dependence and stimulate local job creation. This paper’s analysis confirms these two claims but adds an important nuance: a transition towards a CE can but will not necessarily always result in local job creation or decrease import dependence. The circumstances of the transition towards the CE appear to be of importance. Finally, the analysis identifies the sectors which are indirectly impacted most considerably (in terms of traded volume, demand for labour, input use, etc.). Because of the importance of the indirect impact on other sectors, the aggregate economy-wide effect of the CE measures surpasses the size of the niche repair sector for household appliances considerably.

This paper shows different (fiscal) policy paths for promoting more repair of household appliances, leading towards a more CE. However, the extent to which the policies increase (decrease) the circular (linear) activities differs considerably. On the one hand, expansionary fiscal policies are more effective in the promotion of circular activities compared to restrictive fiscal policies. On the other hand, the restrictive fiscal policies are more effective in reducing the existing linear activities. Policy-makers should reflect on their priorities.

Finally, the uptake of circular activities manages to decrease CO_2_ equivalent emissions from a consumer perspective while it increases these emissions from a production (or territorial) perspective. Hence, the environmental gains are mainly realized at global level since the CE stimulates local economic activities. This also stresses the importance of the selection of the correct perspective while assessing the environmental impact of circular activities. This paper advocates the consumption approach, as this approach provides a more complete picture on a region’s contribution to global emissions. Put differently, the consumption approach accounts for the substitution of less circular international activities by circular local activities. Hence, the strength of the consumption approach in this context is not that it accounts for submissions caused abroad, but that it instead it accounts for emissions avoided abroad. The production approach fails to account for this substitution and hence potentially leads to incorrect conclusions on the environmental impact of circular activities.

### Electronic supplementary material

Below is the link to the electronic supplementary material.

Supplementary file1 (DOCX 69 KB)

## Appendix

### Sectoral labour requirements

Table 5 presents the percentage change in sectoral labour requirements per policy scenario, compared to the baseline scenario. The labour requirements represent the use of one homogenous type of labour, expressed in full time equivalents.

**Table 5:** Percentage change in sectoral labour requirements, per policy scenario

Policy type Sector	Restrictive	Expansionary	Hybrid	Green fiscal reform
Agriculture, fishing, forestry	+ 0.0656	− 0.0011	+ 0.0645	− 5.2337
Mining	+ 0.2929	− 0.0040	+ 0.2890	− 100.0000
Industry	+ 0.0474	− 0.0008	+ 0.0466	− 3.3356
Energy	+ 0.0007	− 0.0010	+ 0.0702	− 4.7599
Construction	− 0.1721	+ 0.00162	− 0.1705	+ 10.6388
Trade	+ 0.0213	− 0.0003	+ 0.0210	+ 1.7106
Land transport	+ 0.0585	− 0.0008	+ 0.0577	− 4.0795
Water transport	+ 0.0768	− 0.0011	+ 0.0757	− 4.4630
Air transport	+ 0.0714	− 0.0011	+ 0.0703	− 3.7100
Logistics & mail	+ 0.0543	− 0.0008	+ 0.0535	− 2.5910
Market services sector	+ 0.0150	− 0.0003	+ 0.0146	+ 2.2748
Non-market services	+ 0.0089	− 0.0001	+ 0.0088	− 0.8815
Retail new household appliances	− 0.9845	− 0.0046	− 0.9891	− 100.0000
Repair household appliances	+ 4.0182	+ 7.9126	+ 12.4308	+ 8.3745

In case of 100 per cent disappearance of labour requirements, this implies a complete shift towards capital services and acquisition of supporting services (e.g. transport, logistics, market services)

### Nested function

Figure 2 presents the generic structure of the consumers’ utility functions. The consumers’ optimize their utility by consuming different products and services. Services and products which are present in the same node in Fig. 2 are substitutes to each other, i.e. the consumer needs to decide upon a consumption basket for that set of products and services.
