# Supplementary material for: Macroeconomic and environmental consequences of circular economy measures in a small open economy
Source: Ann Reg Sci. 2021 Sep 25;68(2):283–306. doi: 10.1007/s00168-021-01079-6 (PMC8475896; doi:10.1007/s00168-021-01079-6)

# Supplementary material

## Basic model’s properties

The most important model properties are listed below:

- Economic agents included in the CGE model: consumers, activities, government, investment, enterprises, the EU and the Rest Of the World (ROW) region;
- A CGE model is a multi-sector model. Each sector is modelled as a representative company with Constant Elasticity of Transformation (CET) production functions. The CET functions are embedded in nested production structures, and calibrated according to Beghin et al. (1996) and van der Mensbrugghe and Peters (2016);
- Profit maximization by firms is subject to labour and capital availability, technology, input of intermediate products (including energy) and services;
- Labour is traded on the labour market and wages are function of the household’s desire for leisure time on the one hand and the interaction between labour demand and labour supply on the other hand. Put differently, the CGE model only captures voluntary unemployment but simultaneously provides insights in the (sectoral and economy-wide) demand for labour;
- Constant Elasticity of Substitution (CES) functions determine consumer decisions. The consumption functions are embedded in nested consumption structures following for example Keller (1975) and Pollak and Wales (1978);
- The available government budget determines government spending. The budget is spent according to a distribution parameter of the government utility function and current consumption prices. The budget available for government spending is variable since it is gathered through taxes on consumption, imports and economic activities. However, the government’s utility level is fixed compared to the baseline scenario. Hence, the available budget for government spending should at least allow to obtain the same government utility as in the baseline situation. In addition, the government deficit should remain constant in real terms;
- Investments depend upon a distribution parameter of the investment utility function and the unit price of investment commodities;
- National income determines aggregate household consumption, public consumption, and savings;
- The exported volume depends upon export prices;
- Domestic and foreign products and services are subject to Armington elasticities as they are not perfect substitutes (Capros et al., 2013);
- The current account is balanced. Hence the total outflow of funds (generated through imports or the payment of net product related taxes to the EU or the ROW) equals the total inflow of funds (generated through exports; net transfers received from the EU or the ROW by governments, households and enterprises; net labour and capital income received from the EU or the ROW);
- All elasticities (including the Armington elasticities) are retrieved out of the technical description of the GEM-E3 model by Capros et al. (2013) except for the consumers’ elasticities of substitution between transport modes which are based on Mayeres (1999);
- The CGE model is complimented with an environmental extension to determine the CO_2_ equivalent emissions per million EUR of spending. These emissions serve as a proxy for the economy’s impact on the climate. The environmental extension uses the standard emission coefficients proposed by Stadler et al. (2018). The tool can calculate the CO_2_ equivalent emissions from both a production (or territorial) perspective and a consumption perspective. The production approach accounts for local emissions due to local consumption and production (independently from the destination of the produced goods and services: local consumption or export). The consumption approach accounts for all emissions which result from the satisfaction of the consumption in a country (independently from the origin of the emissions: local or abroad) (Athanassiadis et al., 2018).

## Visualisation modelling results

The following visualisations do not include the results for the green fiscal reform scenario because the properties of this scenario are too different from the other scenario’s properties: the green fiscal reform directly affects all sectors, while the other policies only directly target two specific sectors. Consequently, the economic impact of the green fiscal reform is considerably higher compared to the other scenarios. Therefore, comparison of all scenarios in a single graph is not useful and would lead to unintelligible figures.

Figure S.1: Percentage change in traded volumes per product and sector following the maximum tax increase or decrease allowed per policy scenario, non-targeted sectors

NOTE.- Non-targeted sectors are all sectors except ‘Household appliances (retail)’, ‘Spare parts household appliances’, and ‘Repair services for household appliances’.

Figure S.2: Change in macroeconomic parameters following the maximum tax increase or decrease allowed per policy scenario, compared to the baseline scenario


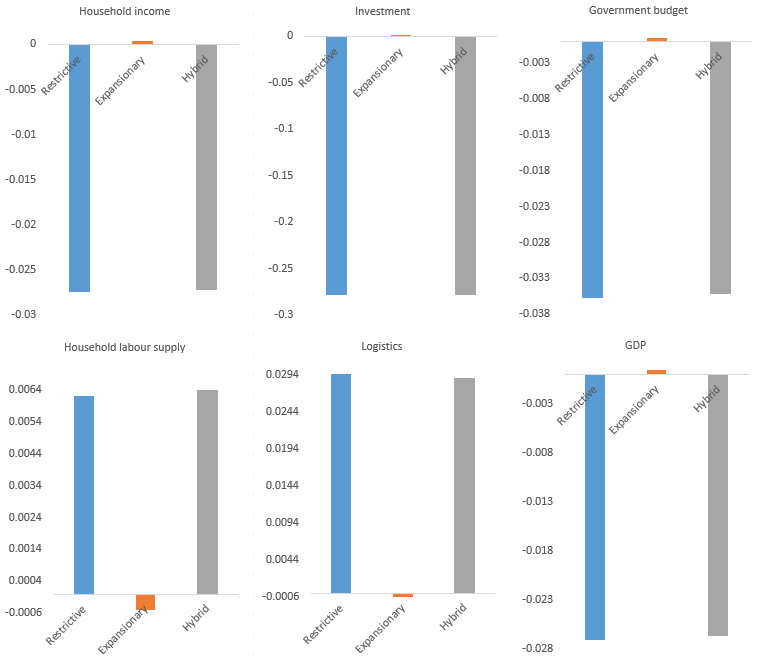

Supplement: Supplementary file 1 — Supplementary file1 (DOCX 69 KB) [file 168_2021_1079_MOESM1_ESM.docx]
